# MXene–MWCNT Conductive Network for Long-Lasting Wearable Strain Sensors with Gesture Recognition Capabilities

**DOI:** 10.3390/mi16020123

**Published:** 2025-01-22

**Authors:** Fei Wang, Hongchen Yu, Xue Lv, Xingyu Ma, Quanlin Qu, Hanning Wang, Da Chen, Yijian Liu

**Affiliations:** Laboratory for Intelligent Flexible Electronics, College of Electronic and Information Engineering, Shandong University of Science and Technology, Qingdao 266590, China; feiwang@sdust.edu.cn (F.W.); yuhc0207@163.com (H.Y.); lxsdkd@163.com (X.L.); mxy18253103525@163.com (X.M.); ququanlin1999@163.com (Q.Q.); wangcc0906@163.com (H.W.)

**Keywords:** Ti_3_C_2_Tx/MWCNT strain sensor, human body detection, machine learning

## Abstract

In this work, a conductive composite film composed of multi-walled carbon nanotubes (MWCNTs) and multi-layer Ti_3_C_2_Tx MXene nanosheets is used to construct a strain sensor on sandpaper Ecoflex substrate. The composite material forms a sophisticated conductive network with exceptional electrical conductivity, resulting in sensors with broad detection ranges and high sensitivities. The findings indicate that the strain sensing range of the Ecoflex/Ti_3_C_2_Tx/MWCNT strain sensor, when the mass ratio is set to 5:2, extends to 240%, with a gauge factor (GF) of 933 within the strain interval from 180% to 240%. The strain sensor has demonstrated its robustness by enduring more than 33,000 prolonged stretch-and-release cycles at 20% cyclic tensile strain. Moreover, a fast response time of 200 ms and detection limit of 0.05% are achieved. During application, the sensor effectively enables the detection of diverse physiological signals in the human body. More importantly, its application in a data glove that is coupled with machine learning and uses the Support Vector Machine (SVM) model trained on the collected gesture data results in an impressive recognition accuracy of 93.6%.

## 1. Introduction

Flexible strain sensors with outstanding biocompatibility, scalability, and real-time ability have developed rapidly over the past few decades [1,2,3]. These sensors possess wide-ranging applications [4,5,6], including physiological signal monitoring [7,8,9], electronic skins [10,11,12], wearable electronic devices [13,14,15], and soft robotics [16,17,18,19]. However, there are still limitations restricting the performance of flexible strain sensors. In order to achieve an ideal flexible strain sensor, researchers have explored various strategies to enhance their performance, in which materials with excellent conductivity and compatibility are taken into account, such as carbon nanomaterials [20,21], metal nanoparticles [22,23], nanowires [24,25], reduced graphene oxide (rGO) [26,27], and MXene [28,29]. Among these materials, MXene, which exhibits excellent mechanical and electrical properties, stands out as a promising candidate for flexible wearable strain sensors [30]. Due to the layered 2D structure, MXenes, especially the most utilized Ti_3_C_2_Tx, could achieve significant resistance variations with high-level sensitivity [31], but their flake-like structures impose limitations on the strain range in flexible sensors. For example, Ti_3_C_2_Tx-based sensors achieve an impressive gauge factor (GF) of 7000, but they are limited by a sensing range of merely 5% [32]. Another strain sensor based on multi-walled carbon nanotubes (MWCNTs) could achieve an extensive sensing range of 200%, but the GF of this particular sensor is relatively low at 29.4 [33]. The distinctive spiral structures of MWCNTs allow them to stretch and recover alongside flexible substrate materials during tension and release to enhance the strain sensing range. A common challenge is that pure materials often possess inherent drawbacks that hinder the simultaneous attainment of elevated sensitivity and an expansive sensing range in strain sensors. Fortunately, the combination of MXene and MWCNTs is a promising approach that exhibits noteworthy enhancements in both sensitivity and sensing range [34]. Another key point for ideal flexible strain sensors is the choice of substrate, which plays a significant role in sensor performance. For example, a flexible strain sensor based on a cotton substrate has a strain range of only 15% due to the limitation of cotton’s own stretching [35]. In sensor design, Ecoflex, which is a silica-like polymer widely used in biomedicine, can also be used as a substrate. It is attached to the surface of the human body and does not produce adverse reactions, indicating that it has good biocompatibility [36,37]. In addition, this good biocompatibility also ensures skin safety during long-term use, wherein the good tensile property can effectively improve the performance of the sensor. Furthermore, structural modifications to the substrate could be employed to further enhance the sensor’s sensitivity, such as foam-structured substrate, multi-layered porous structured substrate, and sandpaper-like Ecoflex substrate [38,39]. Therefore, researchers could exploit new opportunities to overcome the limitations via novel material combinations, substrate selection, and structural design, which could enhance the sensitivity and expand the sensing range effectively.

Several works have focused on these key points for high-performance flexible sensors. However, most research has focused on fabricating monolayers of Ti_3_C_2_Tx-MXene or modifying MWCNTs to improve the sensor’s performance [40]. Although material modification can effectively improve its properties, complex manufacturing methods restrict its rapid development and application. Furthermore, the multi-layer MXene structure exhibits superior capability in maintaining overall structural integrity under strain due to interlayer interactions. In flexible strain sensors, when subjected to stretching, bending, or other deformations, the multi-layer architecture effectively prevents material rupture and excessive deformation. While single-layer MXene may offer enhanced electrical performance, it is comparatively inferior in terms of mechanical stability and durability [41,42]. Moreover, the fabrication method involving multi-layer clay-like Ti_3_C_2_Tx combined with pristine MWCNTs is straightforward. However, detailed analysis of the mechanism of flexible strain sensors on Ti_3_C_2_Tx/MWCNTs and sandpaper-like Ecoflex substrates is scarce. Therefore, it is urgent to systematically study whether the working mechanism of the sensor can improve its performance, and it is equally important to find its potential application fields, which is of great significance for the development of flexible strain sensors in the future.

In this study, we combine Ti_3_C_2_Tx and MWCNTs to create a multi-material system, which was coated on sandpaper-like Ecoflex substrate to fabricate flexible strain sensors. It has been observed that the composite material establishes a sophisticated conductive network, which accounts for the sensor’s broad detection range and high sensitivity. Herein, the sensor with a 5:2 weight ratio of Ti_3_C_2_Tx/MWCNTs exhibits an astonishing 240% sensing range, a high sensitivity (GF = 933) within the 180% to 240% strain range, and outstanding cyclic stability over 33,000 cycles. This flexible strain sensor allows comfortable attachment to the skin, enabling effective human body detection of joint movements, facial expressions, and subtle pulse actions. Moreover, it has been successfully applied to a data glove when coupled with machine learning, achieving 93.6% recognition accuracy for ten different gestures, indicating that it can perform classification and recognition intelligently.

## 2. Experiment

### 2.1. Material

The MWCNTs (diameter = 3–15 µm, average length = 15–30 µm, and >95% purity) were provided by Hongdakang Evolution Technology Co., Ltd., Shenzhen, China. The MXene was provided by Guangdong Foshan Xinxi Technology Co., Ltd., Guangdong, China. The Ecoflex (00–30) was purchased from Smooth-on Co., Ltd., Macungie, PA, USA.

### 2.2. Instruments

The deionized water machine used was UPTA−20 from Lichen Instrument Technology Co., Ltd. (Zhejiang, China), and the ultrasonic cleaner used was JP−080S from Jiemeng Technology Co., Ltd. (Chongqing, China). The 12A spin-coater used was KW−4A from the crown electronic equipment factory. The desktop, electric, constant-temperature, blast-drying oven used was DHG−9123A from Shanghai Jinghong Equipment Co., Ltd. (Shanghai, China).

### 2.3. Preparation of Ecoflex

Ecoflex A and Ecoflex B were combined in a 1:1 ratio in a clean, prepared beaker and stirred thoroughly to ensure a homogeneous mixture. The mixture was transferred to a vacuum pump and subjected to vacuum degassing for 10 min to remove any entrapped air bubbles. Subsequently, the uncured Ecoflex colloid was applied onto a pristine glass slide using a spin-coating technique with 50 rad/min spinning speed for 10 s. Upon completion of the spin-coating procedure, the sample was cured at room temperature for future use.

### 2.4. Fabrication of Ecoflex/Ti_3_C_2_Tx/MWCNT Sensors

**Figure 1:**
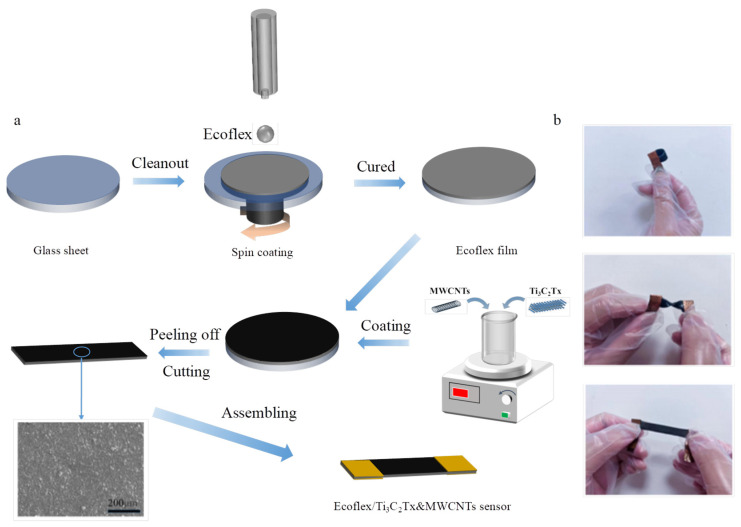
(**a**) The preparation of a Ecoflex/Ti_3_C_2_Tx/MWCNT strain sensor. The insert plots in the lower left corner show the top SEM images of the Ecoflex/Ti_3_C_2_Tx/MWCNT strain sensor at magnifications of 200 μm. (**b**) The folding, twisting, and stretching states of the Ecoflex/Ti_3_C_2_Tx/MWCNT strain sensor.

A mixture of Ti_3_C_2_Tx and MWCNTs was applied onto the surface of Ecoflex after stirring for 24 h using a magnetic stirrer. The composite material was carefully detached from the glass slide and cut into dimensions of 4 cm × 1 cm. Finally, the Ecoflex/Ti_3_C_2_Tx/MWCNT strain sensors were assembled by affixing copper foils, each with a width of 1 cm, to both ends. The experimental groups consisted of four sensors with different conductive materials: pure MWCNT, weight ratios of Ti_3_C_2_Tx:MWCNT = 5:2, weight ratios of Ti_3_C_2_Tx:MWCNT = 10:1, and pure Ti_3_C_2_Tx. All samples were subjected to magnetic stirring at 500 rpm for 24 h to fabricate strain sensors. The entire fabrication process of the Ecoflex/Ti_3_C_2_Tx/MWCNT strain sensor is illustrated in Figure 1a, where the scanning electron microscopy (SEM) images of the strain sensor with a weight ratio of Ti_3_C_2_Tx:MWCNT = 5:2 are presented. The SEM images reveal a uniform incorporation of Ti_3_C_2_Tx material and MWCNTs, where Ti_3_C_2_Tx and MWCNTs were interconnected in an orderly manner, forming a dense sensing layer. The fabricated strain sensor could be observed macroscopically to exhibit foldability, flexibility, and stretchability, as depicted in Figure 1b.

### 2.5. Characterization

The scanning electron microscopy (SEM) images were measured by using the TESCAN MIRA LMS (Brno, Czech Republic). The multifunctional computer-based tensile pressure testing machine (ZHIQU ZQ-990B, Dongwan, China) was used to control the strains applied on the Ecoflex/Ti_3_C_2_Tx/MWCNT strain sensor. The resistance change in the Ecoflex/Ti_3_C_2_Tx/MWCNT strain sensor was recorded with a high-precision digital multimeter (Rigol DM3068 6.5 Digits, Suzhou, China) and a data acquisition instrument (KeySight DAQ970A, Santa Rosa, CA, USA). The calculation formulas for relative resistance change (∆*R*/*R*_0_) and gauge factor (GF) were as follows:

$$\frac{\Delta R}{R0}=\frac{\left(R-R0\right)}{R0}$$

$$GF=\frac{\left(R-R0\right)}{R0\varepsilon }$$

where *R* is the resistance to which strain is applied, *R*_0_ is the initial resistance of the sensor, and ε is the deformation of the sensor.

## 3. Results and Discussion

**Figure 2:**
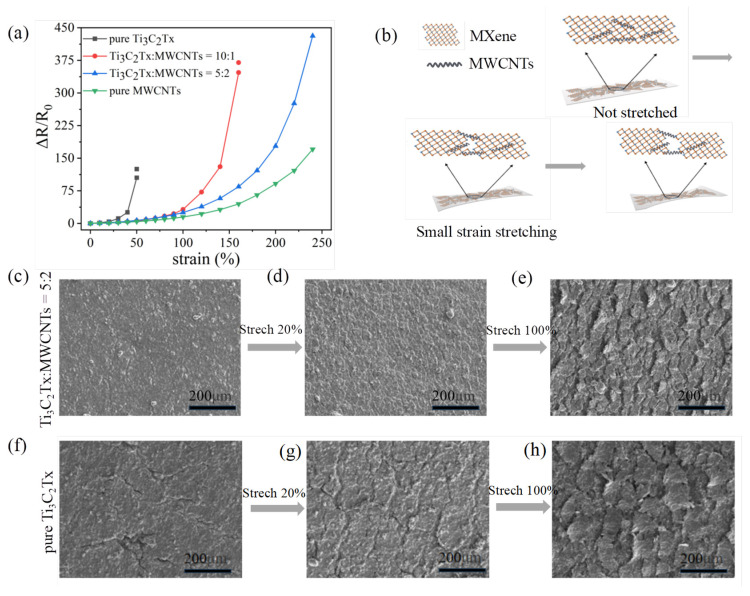
(**a**) The relationship between the resistance change and deformation characteristics of strain sensors with different doping ratios. (**b**) Fracture mechanism of the Ecoflex/Ti_3_C_2_T_x_/MWCNT strain sensor. (**c**–**e**) Top-view SEM images of the Ti_3_C_2_T_x_:MWCNT = 5:2 strain sensor during stretching. (**f**–**h**) Top-view SEM images of the pure Ti_3_C_2_T_x_ strain sensor during stretching.

Figure 2a illustrates the relationship between the relative resistance change and strain obtained through tensile testing for sensors with varying Ti_3_C_2_Tx and MWCNT weight ratios. The pure Ti_3_C_2_Tx sensor has a limited sensing range of only 50%. When the weight ratio of Ti_3_C_2_Tx:MWCNT is 10:1, the strain range of the sensor can reach 160%. When Ti_3_C_2_Tx:MWCNT = 5:2, the strain range is even more than 240%. It can be seen that the content of Ti_3_C_2_Tx significantly affects the maximum strain, which increases with decreasing content of MXene due to the weak van der Waals forces between the rigid MXene nanosheets. Meanwhile, due to the addition of MWCNTs, MXene/MWCNT interfacial interactions promote the formation of a complex conductive network favoring more conductive paths with a wider strain range and higher sensitivity [43,44]. For device sensitivity, it is found that in Figure 2a, the pure MWCNT sensor exhibits a relative resistance change of only 170.71 at the maximum strain, whereas the Ti_3_C_2_Tx:MWCNT = 5:2 sensor demonstrates a relative resistance change of 431.28. Therefore, it is crucial to control the weight ratio appropriately for the highly beneficial composite effect to ensure the device possesses both wide strain range and high sensitivity.

In order to systematically elucidate the mechanism of performance improvement in the Ecoflex/Ti_3_C_2_Tx/MWCNT sensor, the schematic diagram and SEM images are displayed in Figure 2b–h. Figure 2b is plotted to clearly illustrate the fracture mechanism in the sensing layer. It depicts a microscale schematic diagram of the sensor undergoing deformation. Initially, Ti_3_C_2_Tx and MWCNTs are evenly dispersed on the substrate surface. When the sensor undergoes stretching, the Ti_3_C_2_Tx material initiates fracture, resembling the breakup of glaciers. However, due to their smaller size compared to Ti_3_C_2_Tx, the MWCNTs are able to fill the interstitial spaces between different Ti_3_C_2_Tx sheets, effectively serving as a bridge that connects these sheets, thereby furnishing additional conductive pathways. As a result, the device maintains conductivity, but the electrical conductivity gradually decreases during this process. With continued stretching, the interconnections between the Ti_3_C_2_Tx flakes through the MWCNTs gradually decrease, resulting in a rapid decline in electrical conductivity. With further stretching, stripping occurs between Ti_3_C_2_Tx and MWCNTs, resulting in loss of sensing performance. In general, because MWCNTs are effectively connected to Ti_3_C_2_Tx as a filling material, conductive path interconnection is achieved and high performance is obtained, resulting in the strain sensor having a high relative resistance change and sensing range. In order to quantitatively illustrate the conductivity maintained across cracks at various strain levels, the top-down SEM images of Ti_3_C_2_Tx:MWCNT = 5:2 and pure Ti_3_C_2_Tx sensors under different tensile conditions are taken, as shown in Figure 2c–e and Figure 2f–h respectively. It is found that the surface of the Ti_3_C_2_Tx:MWCNT = 5:2 sensor has a dense film at the initial position, while the surface of pure Ti_3_C_2_Tx exhibits the emergence of cracks owing to its flake-like structure. At a strain of 20%, the pure Ti_3_C_2_Tx sensor shows distinct cracking, whereas the cracks on the Ti_3_C_2_Tx:MWCNT = 5:2 sensor remain inconspicuous. Combined with the conductivity in Figure 2a, these results indicate that the MWCNTs act as filler materials across infinitesimal cracks, taking pivotal effect to maintain conductivity. Upon reaching a strain of 100%, small cracks appear on the Ti_3_C_2_Tx:MWCNT = 5:2 sensor, while the cracks on the pure Ti_3_C_2_Tx sensor are further enlarged. Under this circumstance, the MWCNTs serve not only as filler material but also as a bridge that connects the individual Ti_3_C_2_Tx nanosheets. Meanwhile, the Ti_3_C_2_Tx separates entangled MWCNT mutually on account of interfacial interactions [44], which further contributes to the formation of conductive paths to maintain conductivity across cracks. The composite of MWCNTs and Ti_3_C_2_Tx indeed enhances the connectivity of conductive pathways, ensuring that the device not only possesses high sensitivity but also exhibits a broad strain range.

**Figure 3:**
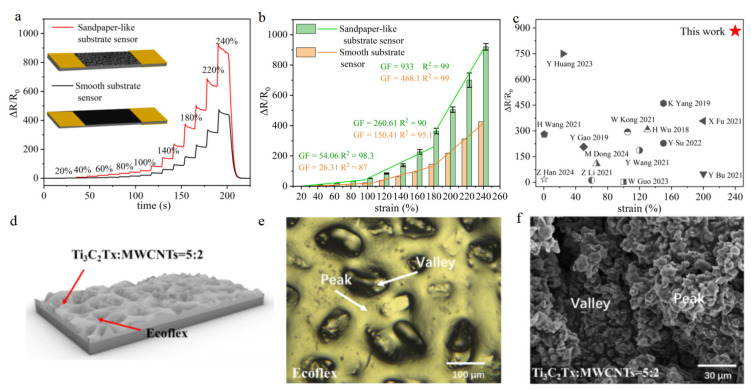
(**a**) Real-time response curves of sandpaper-like substrate and smooth substrate sensors under 0–240% step strain. (**b**) Sensitivities of sandpaper-like substrate and smooth substrate sensors at different strain stages. (**c**) A comparison of the highest gauge factor and the maximum working range of the strain sensors with that of the previously reported strain sensors [45,46,47,48,49,50,51,52,53,54,55,56,57,58]. (**d**) A microscopic diagram of the sandpaper structure. (**e**) A light microscope image of an Ecoflex substrate with a sandpaper structure. (**f**) An SEM image of the surface of the Ecoflex/Ti_3_C_2_Tx/MWCNT sensor with a sandpaper structure.

To further enhance the sensitivity of the strain sensor, structural modifications are implemented on the Ecoflex substrate, incorporating a sandpaper-like microstructure. Overall, through consideration of the good strain range and sensitivity, the Ti_3_C_2_Tx:MWCNT = 5:2 sensor is selected for the subsequent experiment. As shown in Figure 3a, the relative resistance changes of the sandpaper-like structure under segmented tensile testing exhibit significantly higher changes compared with the smooth structure. Figure 3b shows the mean relative resistance changes and the sensitivities of sensors with sandpaper-like substrate and smooth substrate at different strain stages. The average and standard deviation values of five sets of data of each structure are calculated to obtain corresponding bar charts and error bars. It can be seen that the five groups of data are relatively concentrated, avoiding the chance of a single group of data. In addition, it can be seen from Figure 3b that the strain sensor with sandpaper structure has a higher relative resistance change at each strain stage. In the 0–100% strain range, the GF is 54.06. In the 100–180% strain range, the GF is 260.61. In the 180–240% strain range, the GF is up to 933. In addition, the linearity of the three stages is 98%, 90%, and 99%, respectively, indicating that the sensor has good linearity over the three different strain ranges. Compared with previous reports in Figure 3c, our sensor exhibits higher GF (933) and a wider sensing range (240%), which underscores the significant potential of this sensor for applications due to the high sensitivity and the ability to withstand large strains. To elucidate the mechanism of the sandpaper-like structure, the microstructure is depicted in Figure 3d. This microstructure is achieved by introducing a crack zone mechanism to create micro-sized peaks and valleys on the surface of the Ecoflex substrate, thereby complicating the conductive network and further enhancing the device’s sensitivity. An optical microscope image of the Ecoflex surface with the sandpaper-like structure is captured (Figure 3e), which validates the presence of peaks and valleys clearly. Similarly, the SEM image of the strain sensor surface is shown in Figure 3f, where the peaks and valleys are prominently visible. These observations effectively confirm that the sandpaper-like structure effectively complicates the conductive network to enhance sensitivity.

**Figure 4:**
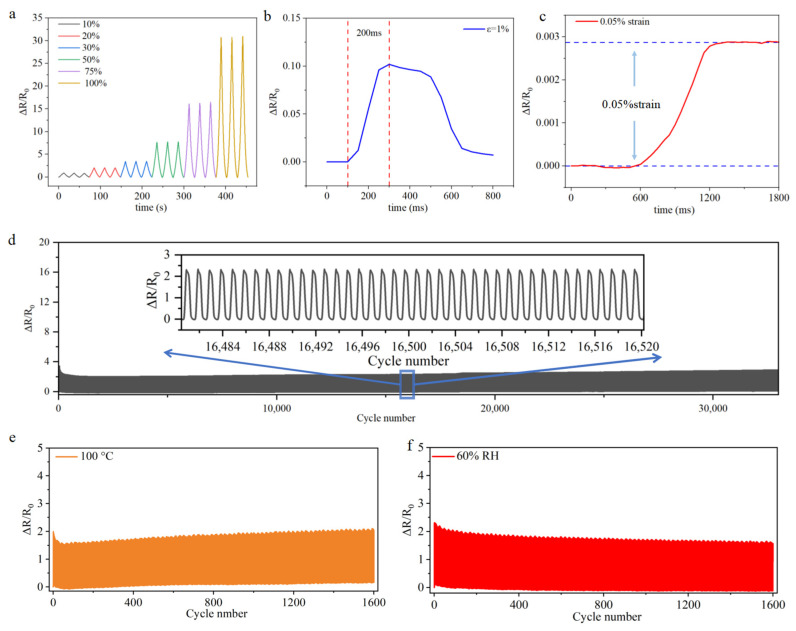
(**a**) The cycling relative resistance variations of the strain sensors with different strains. (**b**) The response time under the strain of 1%. (**c**) The relative resistance changes as a function of time under a minimal strain of 0.05%. (**d**) The long-term durability test of the Ecoflex/Ti_3_C_2_Tx /MWCNT strain sensor with 33,000 stretch-and-release cycles under a 20% strain. The insert plots show the details of the 16,480–16,520 cycles. (**e**) Durability test of the Ecoflex/Ti_3_C_2_Tx /MWCNT strain sensor at 20% strain after heating at 100 °C 1 h. (**f**) Durability test of the Ecoflex/Ti_3_C_2_Tx /MWCNT strain sensor at 20% strain after 2 h at 60%RH.

In addition, further sensing performance measurements are performed on the 5:2 Ti_3_C_2_Tx/MWCNT sensors with sandpaper-like Ecoflex substrates. It is found that in Figure 4a, the sensor consistently exhibits excellent and stable sensing performance during each stage of strain over three cycles at different strains, wherein a wide strain range is obviously achieved. Figure 4b illustrates the relative resistance change of the strain sensor at a strain of 1% and a stretching rate of 500 mm/min. It demonstrates that the strain sensor exhibits a response time of 200 ms, indicating a commendable response rate. This fast response enables the sensor to promptly detect and monitor changes in human physiological signals in real-time. Additionally, when it comes to detecting signals with small strains, the detection limitation becomes an important evaluation criterion, which reaches an impressive value of 0.05% in Figure 4c. Moreover, it is crucial for sensors to possess excellent cyclic stability and durability as these qualities indicate the device’s lifespan. Therefore, conducting tests to assess the cyclic stability of the sensor is performed in Figure 4d. The test results show that the sensor can undergo 33,000 cycles of continuous stretching at a 20% strain, clearly indicating its reliable stability, repeatability, and durability. Furthermore, there are 40 loading–unloading cycles captured between the 16,480th and 16,520th cycles, as shown in the inset in Figure 4d. Notably, the relative resistance change remains insignificant, indicating the sensor’s effective recovery to its initial state after each cycle and its consistent maintenance of sensing performance.

Moreover, durability tests are also performed under high temperatures and in wet environments, in which 1600 cycles are applied to research the sensor performance adequately. Since both the conductive material and the base material are heat-resistant materials, the sensor still maintains good stability and durability at 100 °C under high temperature heating, as shown in Figure 4e. In addition, due to the good packaging of the sensor, it can avoid the adverse effects of a wet environment. When the sensor is subjected to a two-hour exposure in a 60% relative humidity (RH) environment and subsequently evaluated for durability, it continues to exhibit superior stability.

Based on the above analysis, it becomes evident that this Ecoflex/Ti_3_C_2_Tx/MWCNT strain sensor with sandpaper-like Ecoflex substrate possesses outstanding comprehensive sensing performance, including wide strain range, high sensitivity, fast response time, minimal detection limit, and exceptional cyclic stability. Based on the above analysis, it becomes evident that this Ecoflex/Ti_3_C_2_Tx/MWCNT strain sensor with sandpaper-like Ecoflex substrate possesses outstanding comprehensive sensing performance, including wide strain range, high sensitivity, fast response time, minimal detection limit, and exceptional cyclic stability.

Table 1 lists the key parameters of several flexible sensors (including response time, detection limits, and durability). Compared to the above references, the Ecoflex/Ti_3_C_2_Tx /MWCNT strain sensor has a faster response time, a smaller detection limit, and a sensing repeatability of 33,000 times, which are indicative of the superior performance of the sensor in our work and prove the effectiveness of the designed structure.

**Figure 5:**
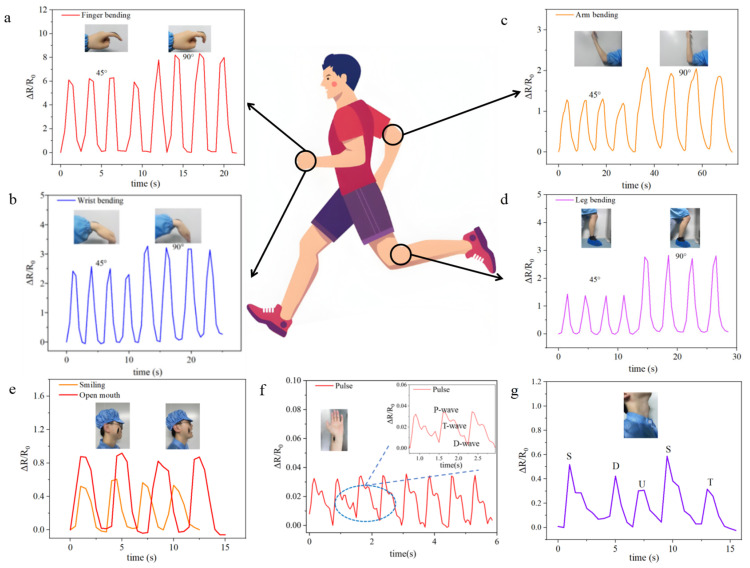
Relative resistance changes in the Ecoflex/Ti_3_C_2_Tx/MWCNT strain sensor attached on the finger (**a**), wrist (**b**), arm (**c**), and leg (**d**). (**e**) Resistance responses of the Ecoflex/Ti_3_C_2_Tx/MWCNT strain sensor in the smiling and open mouth scenarios. (**f**) Pulse signal measured by the Ecoflex/Ti_3_C_2_Tx/MWCNT strain sensor. (**g**) The sensing performance of the Ecoflex/Ti_3_C_2_Tx/MWCNT strain sensor recorded while speaking “S D U S T”.

These qualities render this Ecoflex/Ti_3_C_2_Tx/MWCNT strain sensor highly suitable for practical applications, such as the real-time monitoring of human vital signs and the capturing of minor body movements in wearable flexible sensors. Therefore, the impressive 240% strain range makes it highly effective in detecting joint bending movements. The collected signals accurately capture human body movements and physiological signals, as depicted in Figure 5. Figure 5a–d describes the relative resistance of the index finger, wrist, arm, and leg at flexion angles of 45° and 90°, wherein the results clearly demonstrate the sensor’s ability to accurately differentiate between these different body parts’ degrees of flexion while exhibiting remarkable stability. Notably, significant and regular changes in resistance response are detected at four different joint locations. These results strongly validate the sensor’s capacity to detect major joint bending movements in the human body. Moreover, the sensor exhibits an impressive ultra-low detection limit of 0.05%, which can be effectively used for subtle change or small strain detection in the human body. For instance, Figure 5e shows that the resistance changes regularly and steadily during repeated opening and closing of the mouth, which is measured with the sensor attached to the face. When smiling, the periodic change in resistance can also be clearly detected. Meanwhile, the resistance change while smiling is obviously less than that while opening the mouth, which coincides with the actual smile and mouth opening movement amplitudes. Thus, these types of subtle changes in the human body can be distinguished easily. Additionally, another potential application in healthcare involves small strain detection, which can be placed at the pulse point to monitor pulse signals. Pulse detection is conducted in Figure 5f, where pulse signals are acquired with three waves. The P, T, and D waves are distinguished and consistent with the waveform of the output signals in the relevant study [67], proving the effectiveness of our sensors in healthcare. The detected heart rate from the subject, approximately 75 beats per minute under normal conditions, is closely aligned with the rate detected by the sensor, underscoring the sensor’s high sensitivity and real-time detection capabilities, demonstrating excellent performance even in detecting minute strain variations. Consequently, this sensor holds immense potential for providing rapid and non-invasive diagnostic methods in the healthcare industry. Furthermore, the sensor is positioned on the throat to monitor changes during various pronunciations. The resistance change curves obtained during the pronunciation of “S D U S T” (Figure 5g) clearly distinguish the pronunciation of different letters. Additionally, the resistance change curves for the two instances of pronouncing “S” are nearly identical, indicating that the sensor can not only differentiate between different letter pronunciations but also classify similar pronunciations. This remarkable speech recognition ability of the sensor opens up significant applications in speech rehabilitation training and voice control. These results show that this sensor has a remarkable capability to detect both major joint movements and subtle changes in human body.

**Figure 6:**
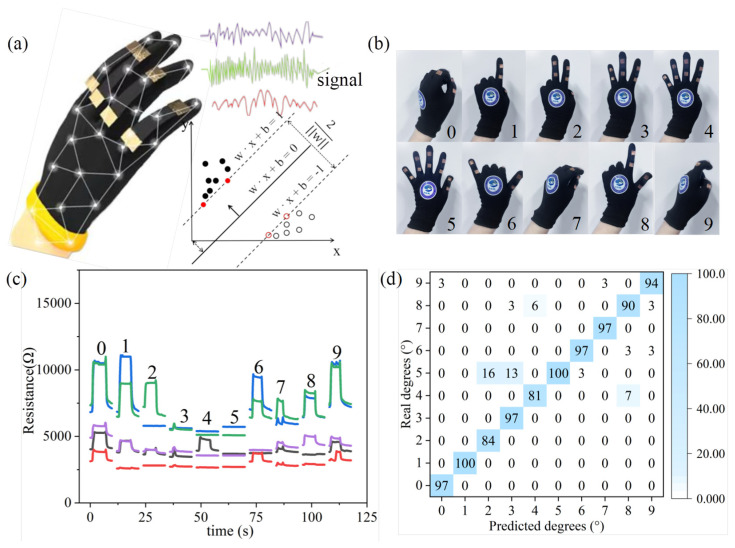
(**a**) A conceptual diagram of the designed data glove. (**b**) An actual image of the data glove displaying “0”–“9,” representing ten different gestures. (**c**) The resistance change waveforms collected for the “0”–“9” gestures. (**d**) The confusion matrix of the prediction results using the SVM model.

Considering the outstanding sensing performance and stability of the sensor, it can also be applied to data gloves assisted by a Support Vector Machine (SVM) model for the classification and recognition of different hand gestures; the diagrammatic sketch is shown in Figure 6a. During the experiment, the strain sensor is first attached to the glove and connected to the KeySight DAQ970A. Subjects make gestures ranging from “0” to “9”, and the ten different gestures are shown in Figure 6b. It is obvious that in Figure 6c, each gesture provides a unique waveform. The collected data are acquired, imported, and sliced. Each signal contains five sets of 50 data points (250 data points) for a total of 1000 signal sets (100 per gesture). Subsequently, the 1000 signals are randomly divided into a training set (700 signals) and a test set (300 signals). To achieve automatic recognition of different gestures, a computationally lightweight SVM model is employed. Feature peaks are segmented using a peak detection algorithm, and machine learning is performed using the simple SVM model. Ultimately, the classification accuracy of the training set reaches 93.6%, which is sufficient for practical applications. The confusion matrix of the SVM model’s prediction results is presented in Figure 6d. The prediction accuracy for each category displays promising results, indicating the effective application potential of the sensor in data gloves, which illustrates that this sensor can be utilized with machine learning for intelligent classification and recognition.

Table 2 lists a comparison of intelligent gesture recognition rates of several flexible sensors. It can be seen that the Ecoflex/Ti_3_C_2_Tx /MWCNT strain sensor is able to show an excellent recognition rate at a high number of gestures (ten) compared to the above references. It shows that the sensor has better potential in intelligent recognition applications.

## 4. Conclusions

In summary, we created a strain sensor incorporating multi-layered Ti_3_C_2_Tx and MWCNTs with a sandpaper-like Ecoflex substrate that exhibited a high sensitivity and wide detection range. The MXene–MWCNT conductive network enhanced the sensors’ performance effectively, wherein 5:2 weight ratio device achieved an impressive GF of 933 in a strain range of 180% to 240% and an impressive wide strain range of 240%. A rapid response time of 200 ms and a detection limit of 0.05% could also be obtained. Furthermore, the sensor demonstrated outstanding stability and sensing performance and remained stable even after 33,000 cycles at 20% strain, with no substantial increment in relative resistance. These remarkable sensing capabilities made the sensor highly suitable for applications involving human motion, including extensive joint movements, facial expressions, and data gloves. Our results revealed the mechanism underlying the high performance in Ti_3_C_2_Tx/MWCNT strain sensors with sandpaper-like Ecoflex substrates. We suggested potential applications of this sensor in human health monitoring, human–machine interaction, speech recognition, and soft robotics, which are significant for the future development of flexible strain sensors.

## Figures and Tables

**Table 1:** Compare the performance of relevant flexible strain sensors (response time, detection limit, and durability).

Composition	Response Time	Detection Limit	Durability	Ref.
CB/PANIP/TPU		0.03%	10,000	[59]
MDS-LIG/PDMS	250 ms		12,000	[60]
PVA/DMSO/rGO/GO	250 ms		4000	[61]
CNTs/rGO/PDA/HF-SIO_2_	200 ms	0.1%	1000	[62]
AgNWs/NFC	180 ms	0.5%	10,000	[63]
CNTs/PVA	508 ms		1000	[64]
PAM/CA/EtOH_30_	340 ms		200	[65]
IL/TPU		0.1%	1000	[66]
Ecoflex/Ti_3_C_2_Tx /MWCNTs	200 ms	0.05%	33,000	This work

**Table 2:** A comparison of the intelligent gesture recognition rates of other flexible strain sensors.

Composition	Number of Gestures	Method	Recognition Rate	Ref.
CNT/graphene/PDMS	10	SVM	91.6%	[68]
PAM/SA/TG	9	1D CNN	83.3%	[69]
GNSs/MWCNTs	5	LSTM	95%	[70]
Graphene	10	MobileNet CNN	90%	[71]
Ionogels	10	DCNN	93.66%	[72]
Graphene/PET	12	SVM	84.7%	[73]
CNTs/graphene/TPU	6	KNN	95.7%	[74]
Ecoflex/Ti_3_C_2_Tx /MWCNT	10	SVM	93.66%	This work

## Data Availability

The data that support the findings of this study are available from the corresponding author upon reasonable request.
